# Nonfatal Firearm Violence Trends on the Westside of Chicago Between 2005 and 2016

**DOI:** 10.1007/s10900-018-00603-8

**Published:** 2018-12-18

**Authors:** Veronica Fitzpatrick, Myles Castro, Jacquelyn Jacobs, Nadew Sebro, Jhoanna Gulmatico, Maureen Shields, Sharon Marie Homan

**Affiliations:** 1 Sinai Urban Health Institute, 1500 S. Fairfield Ave K448, Chicago, IL 60608 USA; 2 Mount Sinai Hospital, Chicago, IL USA

**Keywords:** Epidemiology, Nonfatal firearm violence, Chicago, Nonfatal injury, Hospitalization

## Abstract

This paper examines the epidemiology of nonfatal firearm violence (NFFV) on the Westside of Chicago over three finite time periods: 2005–2008, 2009–2012, and 2013–2016. The trend analysis will look at any significant changes over the time periods and describe the demographic characteristics of NFFV. A descriptive analysis of Mount Sinai Hospital (MSH) Emergency Department (ED) data was conducted. NFFV patients were identified by specific firearm ICD-9 primary eCode injury categories: accident, assault, legal intervention, undetermined intent, suicide or self-inflicted injury, and legal intervention. The Pearson Chi-square test was conducted to statistically compare the categorical frequencies of the Chicago metropolitan region of injury, cause of firearm related injury, and place of injury by time period. There were a total of 3962 nonfatal hospitalizations at MSH between the three time periods due to gun violence related injuries. Overall, nonfatal hospitalizations were most frequent for those between age groups 16–24 (52.5%). The number of nonfatal hospitalizations decreased with increasing age for the age groups 35–44 (9.7%), 45–54 (3.2%), and > 54 (1.2%). There were significantly more nonfatal hospitalizations in males (n = 3649) than females (n = 312) across the three time periods. However, there was a 74.7% increase in female nonfatal hospitalizations from 2009–2012 to 2013–2016. There were significant racial differences in nonfatal hospitalizations between the three time periods. NFFV continues to be problem on the Westside of Chicago, particularly for young, Black men. The incidence of gun violence however has not changed significantly between 2005 and 2016.

## Introduction

The frequency and impact of firearm injury makes it an important public health problem. While many initiatives and programs have been funded to combat the effects of firearm violence, there have been very few places in the United States where firearm violence has decreased or diminished [11]. While much of the public narrative centers on firearm fatalities, the number of nonfatal firearm injuries far exceeds fatalies. Between 2001 and 2013, there were approximately 921,613 nonfatal firearm injuries rates in the United States as compared with 406,946 fatal firearm injuries [13].

The majority of firearm violence victims are not fatally injured, and about 80% of those injured are hospitalized [14]. Hospitals are critical to the firearm violence narrative, as their EDs and inpatient units see the majority of firearm violence victims. Between 2003 and 2013, the annual rate of hospital admissions due to firearm injuries nationally was 30,617 [20]. More than 80% of these hospitalizations were among individuals aged 15–44 [20]. Rates were nine times higher for males than females and nearly ten times higher for Non-Hispanic Black than White individuals. Of the injuries in which the firearm type was known, 70% were from handguns [20]. NFFV injuries cause significant burden on individuals. Those who have been hospitalized for a firearm injury may have to spend additional time in a rehabilitation facility as well as endure substantial morbidity and poor quality of life long after discharge.

Hospital EDs are a critical place to assess the incidence of nonfatal firearm injury since the majority of those injured will have to go through an ED to be treated. Data on firearm injury from the National Electronic Injury Surveillance System (NEISS) shows that between 2010 and 2012, U.S. Emergency Departments (ED) treated 201,591 people with nonfatal firearm injuries [9]. Of these injuries, 72% were under the age of 35, 89% were male, and approximately 37% were Non-Hispanic or Hispanic Black [9]. Long term consequences of NFFV include lost productivity, high medical costs, physical disabilities, such as spinal cord injuries, and chronic mental health problems, such as post-traumatic stress disorder or increased aggression [8, 9, 25]. Lifetime costs for nonfatal firearm injuries are daunting. The total lifetime costs due to a combination of work loss and medical costs of nonfatal firearm injuries equates to over $4.25 billion [9].

Chicago, Illinois is plagued by firearm violence, making it of particular interest to the public media. In 2016, Chicago recorded 764 shooting victims, more than New York and Los Angeles combined [6, 7]. Chicago is made up of 77 community areas that vary significantly in socioeconomics, race, and hardship. Similar to other urban cities in the U.S., Chicago’s firearm violence disproportionately occurs in the most disadvantaged neighborhoods. In order to look at this, we focused on a hospital system serving a particularly disadvantaged neighborhood, disproportionately affected by firearm violence. Research tends to focus on the consequences of fatal firearm injuries, therefore this is an opportunity to better understand the demographic and clinical outcomes from NFFV [13]. This analysis of MSH’s NFFV data will provide a more detailed look at who is affected by firearm violence and how that has changed over time. With a deeper grasp of who experiences NFFV injuries, public health programming will be able to most effectively improve the health and well-being of urban areas plagued by firearm violence [15]. The objective of our study was to assess the temporal trends of nonfatal firearm injury in Chicago, Illinois, using hospitalization data from a Level 1 Trauma Center located in the city’s Westside.

## Methods

### Study Design

To develop a descriptive analysis of NFFV in Chicago, we conducted a secondary analysis of hospital patient data that was extracted from the Illinois Department of Public Health Trauma Registry (IDPHTR). The IDPHTR is a mandatory trauma reporting system for all Illinois trauma centers [21]. We collected IDPHTR data for any MSH patients who were admitted through the ED or treated in the ED for at least eight hours from January 2005 to December 2016. Patients were identified by an ICD-9 primary eCode injury category of accident, assault, undetermined intent, suicide or self-inflicted injury, and legal intervention by firearm (Table 1). Patients were excluded if the record had a discharge status of “morgue”, “funeral home”, “medical examiner”, or “coroner.” Patient data were divided into three time periods: 2005–2008.

**Table 1:** ICD-9 firearm injury eCode categories and respective eCodes

Firearm ICD-9 primary eCodes	
Category	eCodes
Accidents	E922.0; E922.1; E922.2; E922.3; E922.8; E922.9
Assault	E965.0; E965.1; E965.2; E965.3; E965.4
Undetermined intent	E985.0; E985.1; E985.2; E985.3; E985.4
Suicide or self-inflicted injury	E955.0; E955.1; E955.2; E955.3; E955.4; E955.9
Legal intervention	E970

### Study Population

Mount Sinai Hospital is located on the Westside of Chicago in a neighborhood named North Lawndale. North Lawndale, is considered one of the most dangerous neighborhoods, seeing 7% of Chicago’s firearm violence overall [5]. The residents of North Lawndale are primarily Non-Hispanic Black (89%) with a median age of 29; 55% of the population makes less than $25,000 per year [2]. North Lawndale is also home to Mount Sinai Hospital (MSH), a non-profit safety net health system which serves a diverse area of about 1.5 million people. The MSH ED is a Level 1 Adult Trauma Center, providing care to approximately 65,000 patients each year and is one of just four^1^ trauma centers in Chicago. Considering the size of Chicago’s population, and the limited number of Level 1 Trauma Centers serving its most vulnerable communities, it is accurate to say that Mount Sinai sees a large percentage of the total firearm violence that occurs in Chicago.

### Variables

We examined temporal trends in the following variables over the three identified time periods: patient age, gender, race and ethnicity, Chicago metropolitan area region of injury, cause of firearm related injury, and place of injury. Chicago metropolitan area region of injury was determined by scene of injury zip code. Scene of injury zip codes were grouped into Chicago community areas, and the Chicago community areas were further grouped into Chicago metropolitan area regions: Westside, South/Southwest side, and other. We did our best to estimate Chicago metropolitan area regions for regions that crossed over multiple zip codes. The Chicago metropolitan area region that contained the larger area of the zip code retained the zip code’s total population (Table 2). The “Other” category includes zip codes located in the city of Chicago and in its adjacent suburbs [1].

**Table 2:** Chicago metropolitan area zip codes

Chicago metropolitan area zip codes	
Chicago area	Zip codes
Westside	60607; 60608; 60610; 60612; 60622; 60623; 60624; 60639; 60644; 60647; 60651
South/Southwest side	60609; 60615; 60616; 60617; 60619; 60620; 60621; 60629; 60632; 60636; 60637; 60638; 60649; 60652; 60653
Other	60005; 60062; 60104; 60130; 60153; 60155; 60301; 60402; 60409; 60419; 60426; 60429; 60453; 60455; 60461; 60473; 60534; 60563; 60601; 60605; 60611; 60618; 60625; 60626; 60627; 60628; 60630; 60633; 60634; 60640; 60641; 60643; 60648; 60658; 60659; 60664; 60704; 60804; 60805; 60827; 63101

### Analysis

Data was obtained from the IDPHTR and transferred to IBM SPSS Statistics 25 for quantitative analysis. Pearson’s Chi square tests were conducted to compare the distributions of each categorical variable across the three time periods. Age was also assessed as a continuous variable, using one-way ANOVA to test for differences in the mean patient age across the three time periods.

## Results

Between three time periods (2005–2008, 2009–2012, and 2013–2016), there were a total of 3962 nonfatal hospitalizations due to firearm related injuries at MSH (Table 3), (n = 1227), 2009–2012 (n = 1292), and 2013–2016 (n = 1443). The time period of 2013–2016 had the most NFFV hospitalizations (n = 1443), followed by 2009–2012 (n = 1292), then 2005–2008 (n = 1227). The average patient age overall was 25.2 years (SD = 9.3; range < 1–95), with little difference in mean age or distribution of age across time periods. Overall, nonfatal hospitalizations were most frequent among those aged 16–24 (52.5%; 95% CI 50.9, 54.1) and 25–34 (27.5%; 95% CI 26.1, 28.9). The number of nonfatal hospitalizations decreased with increasing age (Table 3).

**Table 3:** Nonfatal firearm violent injury characteristics in three time periods (2005–2008, 2009–2012, and 2013–2016) by age, gender, race and ethnicity, cause of injury, and location

Time period	2005–2008,			2009–2012			2013–2016			Total			*P*
	No		% (95% CI)	No		% (95% CI)	No		% (95% CI)	No		% (95% CI)	
Population size	1227			1292			1443			3962			
Age, years^a^													.934*
Mean	24.9 ± 9.0			25.1 ± 9.2			25.4 ± 9.7			25.2 ± 9.3			.433**
< 16	78	6.4 (5.0, 7.8)		72	5.6 (4.3, 6.9)		82	5.7 (4.5, 6.9)		232	5.9 (5.2, 6.6)		
16–24	649	52.9 (50.1, 55.7)		680	52.6 (49.9, 55.3)		753	52.2 (49.6, 54.8)		2082	52.5 (50.9, 54.1)		
25–34	330	26.9 (24.4, 29.4)		363	28.1 (25.6, 30.6)		395	27.4 (25.1, 29.7)		1088	27.5 (26.1, 28.9)		
35–44	116	9.5 (7.9, 11.1)		122	9.4 (7.8, 11.0)		148	10.3 (8.7, 11.9)		386	9.7 (8.8, 10.6)		
45–54	43	3.5 (2.5, 4.5)		38	2.9 (2.0, 3.8)		44	3.0 (2.1, 3.9)		125	3.2 (2.7, 3.7)		
> 54	11	0.9 (.4, 1.4)		17	1.3 (.7, 1.9)		21	1.4 (0.8, 2.0)		49	1.2 (.9, 1.5)		
Gender													.001*
Male	1143	93.2 (91.8, 94.6)		1209	93.6 (92.3, 94.9)		1297	89.9 (88.3, 91.5)		3649	92.1 (91.3, 92.9)		
Female	84	6.8 (5.4, 8.2)		83	6.4 (5.1, 7.7)		145	10.1 (8.5, 11.7)		312	7.9 (7.1, 8.7)		
Race/ethnicity^a^													< .001*
White	25	2.0 (1.2, 2.8)		22	1.7 (1.0, 2.4)		21	1.5 (0.9, 2.1)		68	1.7 (1.3, 2.1)		
Black	801	65.3 (62.6, 68.0)		920	71.2 (68.7, 73.7)		1093	75.7 (73.5, 77.9)		2814	71.0 (69.6, 72.4)		
Hispanic	369	30.1 (27.5, 32.7)		334	25.9 (23.5, 28.3)		304	21.1 (19.0, 23.2)		1007	25.4 (24.0, 26.8)		
Other^b^	32	2.6 (1.7, 3.5)		16	1.2 (.6, 1.8)		25	1.7 (1.0, 2.4)		73	1.8 (1.4, 2.2)		
Chicago metropolitan area of injury^a^													< .001*
Westside	558	45.5 (42.7, 48.3)		821	63.5 (60.9, 66.1)		938	65.0 (62.5, 67.5)		2317	58.5 (57.0, 60.0)		
South/Southwest side	154	12.6 (10.7, 14.5)		272	21.1 (24.5, 29.3)		335	23.2 (21.0, 25.4)		761	19.2 (18.0, 20.4)		
Other^c^	44	3.6 (2.6, 4.6)		52	4.0 (2.9, 5.1)		64	4.4 (3.3, 5.5)		160	4.0 (3.4, 4.6)		
Unknown	471	38.4 (35.7, 41.1)		147	11.4 (9.7, 13.1)		106	7.3 (6.0, 8.6)		724	18.3 (17.1, 19.5)		
Cause of firearm related injury													< .001*
Accident	76	6.2 (4.9, 7.5)		127	9.8 (8.2, 11.4)		86	6.0 (4.8, 7.2)		289	7.3 (6.5, 8.1)		
Assault	1120	91.3 (89.7, 92.9)		1106	85.6 (83.7, 87.5)		1282	88.8 (87.2, 90.4)		3508	88.5 (87.5, 89.5)		
Undetermined intent	30	2.4 (1.5, 3.3)		53	4.1 (3.0, 5.2)		62	4.3 (3.3, 5.3)		145	3.7 (3.1, 4.3)		
Other^d^	1	0.1 (0.0, 0.3)		6	0.5 (0.1, 0.9)		13	0.9 (0.4, 1.4)		20	0.5 (0.3, 0.7)		
Place of injury													< .001*
Home	69	5.6 (4.3, 6.9)		96	7.4 (6.0, 8.8)		94	6.5 (5.2, 7.8)		259	6.5 (5.7, 7.3)		
Public building	17	1.4 (0.7, 2.1)		19	1.5 (0.8, 2.2)		26	1.8 (1.1, 2.5)		62	1.6 (1.2, 2.0)		
Street and highway	788	64.2 (61.5, 66.9)		1029	79.6 (77.4, 81.8)		925	64.1 (61.6, 66.6)		2742	69.2 (67.8, 70.6)		
Other^e^	353	28.8 (26.3, 31.3)		148	11.5 (9.8, 13.2)		398	27.6 (25.3, 29.9)		899	22.7 (21.4, 24.0)		

*Chi square, **ANOVA

^a^May not add to total due to rounding

^b^Unknown, not classified; Asian or Pacific Islander; Hawaiian; Other Asian or Pacific Islander; other; empty

^c^60005; 60062; 60104; 60130; 60153; 60155; 60301; 60402; 60409; 60419; 60426; 60429; 60453; 60455; 60461; 60473; 60534; 60563; 60601; 60605; 60611; 60618; 60625; 60626; 60627; 60628; 60630; 60633; 60634; 60640; 60641; 60643; 60648; 60658; 60659; 60664; 60704; 60804; 60805; 60827; 63101

^d^Legal intervention; suicide and self-inflicted injury

^e^Other specified place of occurrence; industrial place and premise; recreation and sport; residential institution; mine and quarry; unspecified place of occurrence; empty

Overall, there were more nonfatal hospitalizations among males (92.1%; 95% CI 91.3, 92.9) than females (7.9%; 95% CI 7.1, 8.7). There were significant racial/ethnic differences in nonfatal hospitalizations between the three time periods (p ≤ .001). Overall, the non-Hispanic Black population (71.0%; 95% CI 69.6, 72.4) was most affected by NFFV injuries. Blacks being hospitalized increased from 65% of all visits in 2005–2008 to 75% of all visits in 2013–2016, despite the fact that only 50% of MSH patients overall are Black (Table 3).

Between the three time periods, significant differences in cause of injury (p ≤ .001) and location (p ≤ .001) were noted. NFFV injuries due to assault (88.5%; 95% CI 87.5, 89.5) were most common, and increased by 15.9% from 2009 to 2012 to 2013–2016. Injuries due to firearm related accidents decreased by 32.3% during the same time period, and accounted for 7.3% (95% CI 6.5, 8.1) of total firearm related injuries. The majority of the injuries took place on the Westside of Chicago (58.5%; 95% CI 57.0, 60.0), and on the South/Southwest sides of Chicago (19.2%; 95% CI 18.0, 20.4). Additionally, there was an increase of hospitalizations on the Westside and South/Southwest sides of Chicago by 68.1% and 117.5%, respectively, from the time period 2005–2008 to 2013–2016. Overall, firearm related injuries occurred mostly in the streets and highways (69.2%; 95% CI 67.8, 70.6), and least in public buildings (1.6%; 95% CI 1.2, 2.0) (Table 3).

## Discussion

The trends seen from the following analysis illustrate the impact of NFFV in Chicago, at-large, and potentially in similar urban cities. The results provide a descriptive analysis of NFFV patients presenting to the MSH ED during three unique time periods over the course of 14 years. Our findings suggest NFFV due to assault is higher at MSH compared to national data, there are distinct patterns in NFFV patients by age, gender, race/ethnicity, and where shootings occur, and that NFFV can result in significant loss of quality of life of young people due to resulting disability.

### Assault

Over the course of the 14-year study period, assault was the primary cause of injury (85% of all cases), as well as the primary cause of injury during each unique time period. A 2015 analysis of national firearm injuries and death data found that 72% of NFFV injuries was due to assault, lower than what was found at MSH [9]. The high proportion of these injuries being due to assault mean there is an increased risk of retaliation as well as a higher risk of a subsequent trauma, also known as injury recidivism [4, 10, 16]. A 2017 study of over 10,000 admissions in an urban level I trauma center found that of patients with a violent trauma (blunt assault, stabbing, or gunshot wound), patients admitted for a gunshot wound had a 13.5 times higher odds of mortality compared to blunt assault [17]. Compared to blunt assault and stabbings, patients who experienced gunshot wounds were the only violent injury in which severity of the injury increased with each additional hospital admission [17].

### Demographics

Throughout each time period, over 50% of the NFFV occurred among people ages 16–24, and over a quarter of injuries occurred among those aged 25–34. This study also found that significantly more males were affected by NFFV. Historically, males have been the dominant group affected by NFFV, as our results support [22]. However our analysis also found that females experienced a 72.6% increase in NFFV from 2005 to 2008 to 2013–2016 [18]. One of the suspected reasons why there has been a shift in gender for NFFV is due to changes and usage of social media. “Internet banging” and seeing threats and language from gang affiliated individuals online are leading to increased crime and retaliation, which is different from how assault used to manifest—which was primarily in the streets [19].

Compared to other racial and ethnic groups, only the Black population experienced an overall increase of NFFV injuries from 2005–2008 to 2013–2016. Despite large declines within the Black population on the Westside of Chicago, there was still a 36.5% increase in NFFV injuries. Between the years 2005 and 2015, there was a decline of over 100,000 people in the Black population throughout Chicago compared to minor growths within the White, Hispanic, and Asian populations [3]. This can be partly attributed to the decrease in the overall Chicago population and increase exposure to high risk social networks. The city of Chicago has experienced a 6.9% decrease in population between the years 2000 and 2010. During the same time period, communities in West and South/Southwest sides have experienced the majority of these decreases. The Englewood community of the Southwest side experienced a 23.8% decrease in population and the West Garfield Park community of the West side experienced a 21.8% decrease in population.

While firearm violence is prevalent in communities such as those on the South and Southwest sides of Chicago, research suggests that firearm violence and victims of firearm violence are actually quite concentrated within specific populations [24]. A 2012 study of the relationship between gunshot victims and characteristics of their social network found that associating with gang members and knowing others who have been victims of firearm violence significantly increase one’s own chance of becoming victims themselves. Dense high risk social networks are comprised of a small percentage of the community population, but are involved in a majority of community firearm violence [9, 18].

### Community Area/Location

More broadly, violent crime in Chicago on the Westside is comparable to that on the Southside and the Southwest side [1]. However during each of the three year periods, a higher proportion of NFFV injuries at MSH took place on the Westside of Chicago. While citywide, the rates of violence between regions of the city are similar, there are differences in cases presenting to MSH. This is likely due to the location of MSH and its primary service areas [3]. Although MSH’s primary service area stretches into the Southwest side of Chicago, its central location in the Westside of Chicago lends itself to serving more patients from that area.

This MSH hospital data analysis found an increase in injuries on Chicago’s Westside from 512 injuries in 2005–2008 to 894 in 2013–2016. While this increase is quite high, we also see a pretty large decrease in injuries during this time period within those categorized as unknown Chicago Metropolitan area (from 471 injuries to 106 injuries). Although violence on the Westside has increased since 2005, it is just as likely that MSH improved their ability to collect accurate information during patient intake.

### Study Strengths

This study had several strengths. Most importantly this study was able to look at temporal trends of gun violence in a heavily impacted area of Chicago, IL and the ramifications of such in a Level 1 Trauma Center hospital setting. Because this study took place in a hospital, we had access to the data and to a trauma team that was able to explain some of the findings to us. This study is important because we can take some of our findings and begin to see where some potential areas for intervention.

### Study Limitations

This study had several limitations. Our primary limitation is that the Illinois Department of Public Health Trauma Registry (IDPHTR) database only accounts for patients who were in the ED (or subsequently admitted to the hospital) for more than 8 hours. This would imply that there’s an underestimation of the total number of NFFV injuries since a portion of patients are shot and in the hospital for less than 8 hours and would not be counted in the overall totals. Secondly, this study only looked at one urban Level 1 Trauma Center on the Westside of Chicago. Although MSH’s primary and secondary service area covers approximately 1.5 million people, this may not be representative of NFFV on the Southside or the Northside of Chicago. Lastly, there is always an implied limitation when data relies on ICD classifications. Advantageously, this study used ICD-9 coding throughout the three time periods, however this does not mean that the coding could not have been misclassified (e.g. assault when it was an accident).

## Conclusion

Our analysis of 14 years’ worth of nonfatal firearm data from MSH suggests that the most effective public health programming should address Black males between the ages of 16–24. Entry into the ED and further admission to the hospital for NFFV presents an opportunity for intervention during a time when the patient might be most susceptible. Many hospitals do not have protocols or strategies in place to interrupt the cycle of violence, and patients injured by firearms are typically discharged without any screening or intervention taking place. Introducing Hospital-based Violence Intervention Programs (HVIP) can lower injury recidivism, increase medical care payments, and increase overall cost effectiveness [12], Smith et al. [23]. HVIP focuses on reaching high-risk individuals who have been recently admitted to a hospital for treatment of violent injury [23]. Hospitalizations present a “teachable moment” when someone may be open to positive intervention.

## Footnotes

1 As of the time of data collection, there were four Level 1 Trauma Centers in Chicago. By the time of publication there will be five Level 1 Trauma Centers.
